# Circulating tumor DNA for minimal residual disease and recurrence surveillance in hepatocellular carcinoma: current evidence and a translational roadmap

**DOI:** 10.3389/fonc.2026.1920559

**Published:** 2026-09-01

**Authors:** Yang Gu, Xiaofeng Luo, Yuxian Zhang, Jie Tang, Zhigang Wang

**Affiliations:** Department of Hepatobiliary and Pancreatic Surgery, Ward I, Jingmen Central Hospital, Jingmen Central Hospital Affiliated to Jingchu University of Technology, Jingmen, Hubei, China

**Keywords:** cell-free DNA, circulating tumor DNA, hepatocellular carcinoma, liquid biopsy, methylation, minimal residual disease, recurrence surveillance, translational oncology

## Abstract

Recurrence remains a major cause of failure after curative-intent therapy for hepatocellular carcinoma (HCC), including resection, ablation, and liver transplantation. Current surveillance relies on imaging and serum biomarkers, but radiologic relapse may lag behind biological recurrence. This gap has focused attention on circulating tumor DNA (ctDNA) and related cell-free DNA (cfDNA) approaches as candidate tools for minimal residual disease (MRD) assessment and molecular surveillance. In HCC, however, recurrence is biologically heterogeneous. Post-treatment relapse may arise from residual malignant clones, occult intrahepatic spread, or *de novo* hepatocarcinogenesis in a chronically injured liver. This complexity shapes assay selection, result interpretation, and the need for multimodal risk assessment. Tumor-informed ctDNA assays are best suited to tracking residual clonal disease, whereas methylation-based and composite cfDNA approaches may also capture low-shedding disease and recurrence-prone liver-field biology. Available studies suggest that postoperative ctDNA positivity, persistent serial positivity, rising molecular burden, and selected high-risk methylation signals are associated with recurrence risk after curative-intent therapy. Yet the evidence base is uneven across treatment settings and assay classes, laboratory methods are not yet standardized, and binary detectable-versus-undetectable reporting is rarely sufficient for clinical interpretation. The central translational question is therefore how ctDNA/cfDNA information should be used in realistic HCC surveillance pathways. Current evidence supports these assays as adjunctive molecular risk signals, not as standalone treatment triggers. This review synthesizes the evidence, compares assay strategies, outlines multimodal implementation barriers including standardization and cost, and proposes a cautious roadmap for ctDNA-guided recurrence surveillance in HCC.

## Highlights

- HCC recurrence after curative-intent therapy reflects both residual malignant disease and multicentric *de novo* hepatocarcinogenesis within a chronically injured liver.
- Tumor-informed ctDNA is best suited to tracking residual clonal disease. Methylation-based and composite cfDNA assays may also capture low-shedding disease and field-effect risk.
- Post-treatment ctDNA positivity, persistent serial positivity, and higher molecular burden are linked to recurrence risk. Binary positivity alone is not sufficient in all cohorts.
- ctDNA should be interpreted with imaging, AFP/DCP dynamics, pathology, treatment modality, and liver disease background. Implementation also requires standardized laboratory workflows and cost-aware escalation rules.
- Future HCC trials should test whether ctDNA-guided surveillance intensity, imaging timing, and adjuvant trial enrichment improve patient outcomes.

## Methods

This focused translational review was based on searches of PubMed, Web of Science, and major society guidelines. Searches were conducted from January to June 2026 and updated during revision in August 2026. Search terms included “hepatocellular carcinoma”, “HCC”, “circulating tumor DNA”, “ctDNA”, “cell-free DNA”, “cfDNA”, “minimal residual disease”, “MRD”, “recurrence”, “postoperative surveillance”, “ablation”, “liver transplantation”, “methylation”, “fragmentomics”, “circulating tumor cells”, and “exosomes”. We prioritized HCC-specific guidelines, prospective or well-characterized observational studies, systematic reviews, meta-analyses, and recent translational reports. In total, 77 full-text articles or guidelines were reviewed. Twenty-three records were not retained for citation because they were diagnostic-only without recurrence or surveillance relevance, not HCC-specific, preclinical only, focused on non-cfDNA liquid biopsy analytes without direct relevance to the review framework, or duplicated evidence covered by more recent or more directly relevant sources. Fifty-four sources were retained for the final narrative synthesis. The review is a focused narrative synthesis rather than a formal systematic review.

## 1 Introduction

Hepatocellular carcinoma (HCC) remains a major cause of cancer-related mortality. Liver resection, local ablation, and liver transplantation are established curative-intent options for selected patients (1–3). Durable cure is still limited by recurrence. Complete radiologic response or R0 resection does not always mean biological eradication. Many patients later develop intrahepatic or extrahepatic relapse (4).

HCC recurrence is difficult to interpret because it is not a single biological event. Some relapses reflect occult residual malignant disease present at the time of treatment (4, 5). Others arise from continued hepatocarcinogenesis in a chronically injured liver (6). Cirrhosis, chronic viral hepatitis, metabolic liver disease, fibrosis, inflammation, and regenerative nodules create a carcinogenic field. New malignant clones can emerge even after the index tumor has been removed or destroyed (7). Post-treatment risk is therefore shaped by both tumor biology and liver-field biology.

Conventional surveillance remains essential. Cross-sectional imaging and serum biomarkers are central to follow-up. These biomarkers include alpha-fetoprotein (AFP), AFP-L3, and des-gamma-carboxy prothrombin (DCP/PIVKA-II) (8). Yet imaging detects recurrence only after a lesion becomes visible. Serum biomarkers are also not expressed by all tumors (9). The gap between biological recurrence and radiologic relapse remains clinically important.

The early post-treatment period is a key risk window. Early recurrence is associated with aggressive tumor biology, microvascular invasion, occult intrahepatic spread, and poorer survival (10). This window may guide surveillance intensity, adjuvant trial enrichment, and postoperative treatment strategies (11).

Liquid biopsy may help interrogate this hidden phase. ctDNA is attractive because it provides a dynamic blood-based readout of tumor-derived genomic and epigenomic material (12). Broader cfDNA features may add further information. These include methylation patterns, copy-number changes, and fragmentomic signals (13).

**Figure 1:**
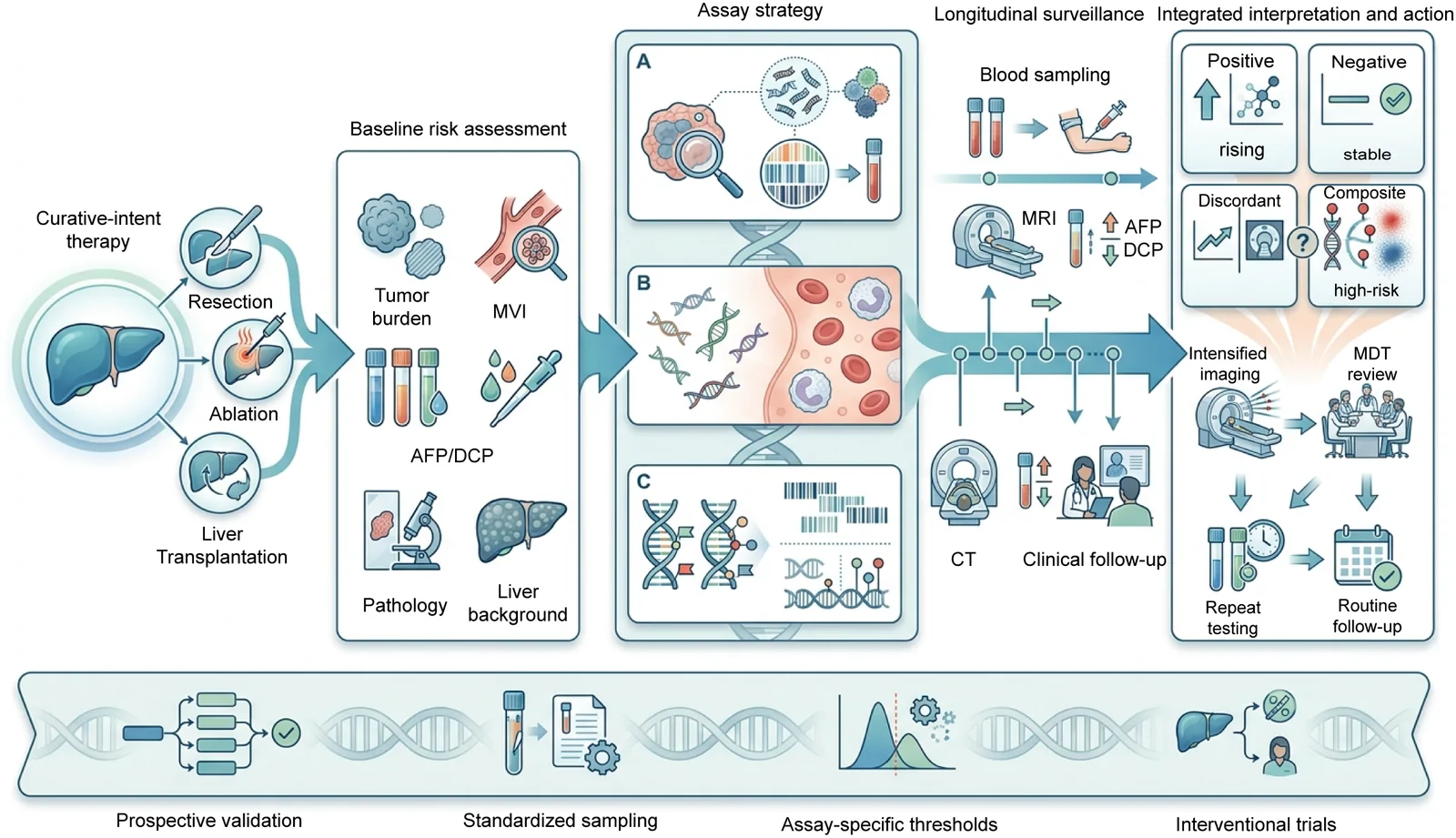
HCC recurrence biology and cfDNA signal sources after curative-intent therapy. This conceptual schematic illustrates how residual malignant disease, intrahepatic metastatic spread, and *de novo* hepatocarcinogenesis in a chronically injured liver may each contribute to recurrence risk and generate partly distinct plasma cfDNA signals. The lower modules represent residual disease, intrahepatic metastasis, and liver field-effect or *de novo* carcinogenesis. Tumor-informed ctDNA assays are best suited to tracking patient-specific residual clonal disease, tumor-naive mutation assays offer scalable plasma detection but are vulnerable to low tumor fraction and clonal hematopoiesis, and methylation, fragmentomic, or composite cfDNA approaches may capture low-shedding disease and recurrence-prone liver-field biology. Positive or rising molecular signals support higher recurrence risk, whereas negative results do not exclude undetected residual disease or later *de novo* tumor formation. Molecular findings should therefore be interpreted with imaging, AFP/DCP, pathology, treatment modality, and liver disease background. AFP, alpha-fetoprotein; AUC, area under the receiver operating characteristic curve; cfDNA, cell-free DNA; CH, clonal hematopoiesis; CT, computed tomography; ctDNA, circulating tumor DNA; DCP, des-gamma-carboxy prothrombin; HCC, hepatocellular carcinoma; LT, liver transplantation; MDT, multidisciplinary team; MPM-8G, methylation predictive model based on eight genes/miRNAs; MRI, magnetic resonance imaging; MRD, minimal residual disease; MVI, microvascular invasion; post-op, postoperative; pre-op, preoperative; TMB, tumor mutational burden.

HCC is not a straightforward MRD setting. Frameworks developed in colorectal or lung cancer cannot be transferred directly because plasma tumor shedding may be limited and the cfDNA background is shaped by cirrhosis, inflammation, hepatocyte injury, and treatment-related tissue damage (14). Tumor-informed assays are well suited to tracking residual clonal disease, but they may miss later *de novo* tumors arising from the injured liver field. By contrast, methylation-based and composite cfDNA approaches may capture broader recurrence-prone biology, although such signals do not necessarily indicate residual disease from the index lesion. **Figure 1** summarizes this interpretive framework.

The clinical challenge is therefore not simply whether ctDNA is detectable, but how a molecular signal should be interpreted in context. That context includes recurrence biology, assay design, sampling time, imaging, serum biomarkers, pathology, treatment modality, and the underlying liver disease background. At present, ctDNA is best viewed as an adjunctive molecular risk signal that may refine surveillance, rather than as an automatic trigger for treatment.

Against this background, this review is organized around the practical steps required for translation. We first define HCC recurrence biology and the rationale for molecular surveillance, then compare mutation-based, tumor-informed, tumor-naive, methylation, fragmentomic, copy-number, and composite cfDNA strategies. We next synthesize evidence across resection, ablation, and transplantation, propose an adjunctive multimodal surveillance framework, and discuss laboratory standardization, cost, and trial requirements before clinical adoption.

## 2 Recurrence biology and the rationale for molecular surveillance in HCC

### 2.1 Recurrence after curative-intent therapy is biologically heterogeneous

Recurrence after curative-intent HCC therapy reflects overlapping processes. Residual malignant disease may persist as microscopic intrahepatic metastases, vascular tumor emboli, or extrahepatic micrometastases (4, 5), whereas multicentric *de novo* carcinogenesis can arise from a chronically injured liver field (6). These mechanisms may coexist, but they have different implications for molecular surveillance.

The early-versus-late recurrence distinction is useful but imperfect. Early relapse is often interpreted as residual disease or occult dissemination, whereas late relapse is often attributed to *de novo* tumor formation (10). Timing alone cannot define mechanism. A postoperative plasma signal matching the treated tumor supports residual clonal disease, especially when it persists across serial samples. Conversely, absence of the original tumor signal should be interpreted as lack of detectable tracked disease, not as proof of cure.

### 2.2 HCC-MRD is not a direct copy of MRD in colorectal or lung cancer

MRD has different meanings across solid tumors. In colorectal and lung cancer, postoperative ctDNA is often interpreted as evidence of residual malignant cells from the treated tumor (15, 16). This logic is also relevant in HCC, particularly after resection of tumors with vascular invasion or other high-risk features, but it is complicated by the persistent carcinogenic liver field (6).

A positive tumor-informed result after resection is conceptually specific for residual disease from the index tumor. A negative result has a narrower meaning: the tracked clone is not detectable in plasma at that time. It does not exclude low-shedding residual disease, sampling below the assay limit of detection, or later multicentric tumor formation (17).

Broader cfDNA approaches answer a related but different question. Methylation signatures, copy-number patterns, fragmentomic profiles, and composite cfDNA models may capture risk beyond the original clone (18). In HCC, these approaches should be treated as complementary to tumor-informed ctDNA rather than as interchangeable tests.

A practical HCC-MRD framework should therefore separate three questions: whether residual disease from the treated tumor is detectable, whether a broader blood-based signal indicates recurrence-prone biology, and whether serial molecular change should alter surveillance intensity.

### 2.3 The cirrhotic and inflamed liver creates a complex cfDNA background

The cirrhotic and inflamed liver adds a second layer of signal complexity. Necroinflammation, fibrosis, vascular remodeling, regenerative activity, and treatment-related tissue injury can increase non-tumor cfDNA and dilute tumor-derived fragments (14, 20, 21). Because residual disease after curative-intent therapy is often low volume, tumor fraction may sit near the limit of detection; therefore, a single early negative or positive result should be interpreted cautiously (17, 19).

For clinical use, this background argues for serial interpretation rather than one-off binary reporting. Persistent positivity, conversion to positivity, or rising molecular burden is more persuasive than isolated early positivity, especially when concordant with imaging, AFP/DCP dynamics, pathology, and treatment context.

### 2.4 Biological rationale for ctDNA- and cfDNA-based postoperative surveillance

ctDNA is the tumor-derived fraction of plasma cfDNA and may reflect changes in tumor burden faster than imaging because cfDNA has a short half-life (12, 13). Persistent tumor-derived DNA after treatment may therefore indicate residual disease before radiologic relapse. HCC studies support this rationale by linking postoperative positivity, persistent perioperative positivity, and quantitative molecular burden with recurrence risk, while also showing that serial dynamics are more informative than isolated detection.

This biology supports a risk-updating model rather than a single-test MRD label. Tumor-informed mutation tracking is best suited to residual clonal disease, whereas methylation and composite cfDNA models may better capture low-shedding disease and field-effect risk. In practice, either result must be interpreted inside a multimodal framework.

## 3 ctDNA and cfDNA assay strategies for HCC-MRD

Assay selection determines which biological signal is measured and how confidently it can be attributed to tumor-derived DNA. In HCC, a plasma sample may contain DNA from residual malignant clones, emerging new tumors, injured hepatocytes, inflammatory cells, and hematopoietic clones. Mutation-based ctDNA assays are a broad molecular category, whereas tumor-informed and tumor-naive designs describe whether plasma variants are anchored to prior tumor sequencing. **Table 1** compares the main assay strategies and their best-fit use cases.

**Table 1:** Comparison of ctDNA/cfDNA assay strategies for postoperative MRD assessment and recurrence-risk surveillance in HCC.

Assay strategy	Main biological signal	Strengths	Limitations	Best-fit HCC use case	Current readiness
Tumor-informed mutation tracking	Patient-specific variants from the treated tumor	High specificity for residual clonal disease; strong biological interpretation when positive	Requires tumor tissue; longer workflow; may miss *de novo* tumors unrelated to the index clone	Post-resection or post-transplant MRD assessment when tissue is available	Translationally advanced but not routine standard of care
Tumor-naive mutation panel	Predefined plasma mutation panel without patient-specific tumor tissue	Tissue-independent; scalable; feasible in retrospective or tissue-limited settings	Lower specificity at low tumor fraction; vulnerable to clonal hematopoiesis without matched leukocyte filtering; not biologically equivalent to tumor-informed tracking	Broad postoperative monitoring when tissue is unavailable	Early translational use
Methylation-based assay	HCC-associated CpG methylation patterns	Relevant in low-shedding disease; may capture field-effect and *de novo* recurrence risk	Biological source of signal may be less direct; thresholds require validation	Recurrence-risk classification, low-shedding surveillance, longitudinal monitoring	Translationally emerging to advanced
Fragmentomics	cfDNA fragment length, end motifs, and nucleosome-related patterns	Does not require known tumor mutation; may add tissue-of-origin information	Limited recurrence-specific validation; analytical pipelines not standardized	Complementary feature in composite cfDNA models	Exploratory
Copy-number analysis	Genome-wide or focal plasma copy-number alterations	Captures broad tumor-derived genomic imbalance; may add prognostic information	Requires sufficient tumor fraction; MRD specificity remains uncertain	Composite recurrence-risk modeling	Exploratory
Composite cfDNA model	Integrated mutation, methylation, fragmentomic, copy-number, and clinical features	Matches biological complexity of HCC; may improve discrimination	Requires large training and external validation cohorts, population calibration, transparent reporting, and standardized laboratory workflows	Adaptive surveillance and risk-updating frameworks	Translationally emerging
Quantitative ctDNA burden or ctDNA-derived TMB	Quantitative plasma molecular burden	Moves beyond binary positivity; may improve postoperative risk stratification	Requires adequate tumor fraction; cutoffs not standardized	Risk enrichment after curative-intent therapy	Exploratory to early translational

MRD, minimal residual disease; HCC, hepatocellular carcinoma; TMB, tumor mutational burden. Readiness categories reflect the current translational maturity of each approach and are narrative judgments rather than formal evidence grades.

### 3.1 Mutation-based ctDNA assays

Mutation-based assays identify somatic variants in plasma. Common platforms include targeted sequencing, digital PCR, and hybrid-capture panels (25). In HCC, recurrently altered pathways include TERT promoter activation, TP53 mutation, CTNNB1 activation, chromatin remodeling, cell-cycle regulation, and receptor tyrosine kinase or RAS signaling (26). This category includes both tumor-informed assays and tumor-naive panels: “mutation-based” defines the molecular signal, whereas “tumor-informed” or “tumor-naive” defines the assay design and the strength of biological attribution.

The main strength is specificity. A tumor-associated mutation, detected in the right clinical context, links the plasma signal to malignant disease. The main limitation is sensitivity in low-volume postoperative disease. A negative result may reflect insufficient tumor fraction rather than absence of residual disease.

Mutation panels also have coverage limits. HCC is heterogeneous, and fixed panels may miss relevant clones. A *de novo* recurrent tumor may not share mutations with the original tumor. Mutation-based assays are therefore strongest for tracking known residual disease. They are weaker for estimating long-term field-driven risk.

### 3.2 Tumor-informed assays

Tumor-informed assays first profile the treated tumor. They then track patient-specific variants in plasma. This design asks a precise question: is DNA from the original tumor still detectable after therapy? Persistence or re-emergence of the same variants strongly supports residual or recurrent clonal disease (27, 28).

The advantage is specificity. Tumor-informed testing anchors plasma analysis to tumor tissue. This reduces ambiguity and helps distinguish tumor-derived signals from background variants, especially at very low variant allele fractions (27). It may be particularly useful after liver transplantation. In that setting, re-emergence of a patient-specific tumor signal is less likely to reflect ongoing native-liver carcinogenesis (28, 29).

The tradeoff is operational complexity. Tumor-informed testing requires adequate tissue, high-quality sequencing, assay customization, and turnaround time. It may be difficult after ablation-only treatment or when archival tissue is limited. It also does not fully address *de novo* recurrence from the liver field.

### 3.3 Tumor-naive mutation panels

Tumor-naive mutation panels are not a separate molecular class from mutation-based ctDNA assays; they are a plasma-only implementation of mutation-based testing. They analyze plasma without prior tumor profiling and use predefined panels that cover recurrently altered genes in cancer or HCC (25). This design is scalable and useful when tissue is unavailable, after ablation-only treatment, or in retrospective cohorts (26).

The tradeoff is interpretive uncertainty. Because the assay is not anchored to patient-specific tumor tissue, a low-frequency plasma variant may arise from tumor, clonal hematopoiesis, or another source. This is especially problematic without matched leukocyte sequencing. Clonal hematopoiesis can generate low-frequency variants that overlap with cancer panels (30, 31).

Accordingly, tumor-naive panels are best viewed as scalable monitoring tools and hypothesis-generating MRD adjuncts rather than definitive proof of residual disease. Their value improves when combined with leukocyte filtering, serial sampling, quantitative thresholds, serum biomarkers, and imaging context.

### 3.4 Methylation-based assays

Methylation-based assays are highly relevant to HCC because aberrant methylation occurs early in hepatocarcinogenesis and can be detected in tissue and plasma (22). Compared with mutation-only approaches, methylation assays interrogate recurrent epigenomic patterns and may remain informative when tumor fraction is low (23).

Their appeal in HCC is twofold. They may improve sensitivity in low-shedding disease (24), and they may capture recurrence-prone biology beyond the original clone, including field-effect risk and *de novo* recurrence susceptibility (32).

Interpretation still requires caution. A high-risk methylation signal may indicate tumor-derived DNA, field-effect biology, or both. Its near-term role is best framed as recurrence-risk classification, longitudinal monitoring, and selection for closer surveillance, not as proof of residual disease from the index tumor.

### 3.5 Fragmentomics, copy-number alterations, and other cfDNA features

Fragmentomics and copy-number analysis extend liquid biopsy beyond variants and methylation. Fragmentomics examines fragment length, end motifs, nucleosome footprints, and genome-wide fragmentation patterns, whereas copy-number analysis evaluates chromosomal gains, losses, and focal amplifications in plasma cfDNA (13, 18, 25). These methods may add information when tumor fraction is too low for robust mutation detection.

Their appeal is breadth: they can capture tumor-derived or tissue-of-origin information without prior knowledge of patient-specific mutations and can be integrated with mutation and methylation data. This is relevant in HCC because tumor shedding is variable and recurrence biology is heterogeneous.

Their limitation is maturity. These features require larger HCC-specific validation cohorts, standardized pipelines, predefined thresholds, and longitudinal recurrence-focused studies. For now, they are best considered components of composite cfDNA models.

### 3.6 Composite cfDNA models

Composite cfDNA models integrate multiple molecular layers, including mutation, methylation, fragmentomics, copy-number alterations, and clinical variables. This approach fits HCC biology because no single signal captures all recurrence mechanisms (13, 25).

Tumor-informed mutation tracking can identify residual disease from the index tumor, while methylation and fragmentomic features may add information about low-shedding recurrence or field-associated risk. Clinical variables such as microvascular invasion, tumor size, AFP/DCP dynamics, cirrhosis, and treatment modality can further refine interpretation (8, 33).

Composite models also introduce complexity. Performance depends on model training, external validation, population calibration, and transparent reporting. HBV-, HCV-, alcohol-, and metabolic liver disease-associated HCC may differ in tumor biology and cfDNA background, so validation across etiologies and treatment settings is required.

### 3.7 Pre-analytical and analytical considerations

Pre-analytical and analytical standardization is a central barrier to comparing HCC-MRD studies. The expected signal is often close to the technical limit of detection, so blood collection tubes, time to plasma separation, centrifugation, plasma volume, cfDNA extraction, cfDNA input, sequencing depth, molecular barcoding, error suppression, and bioinformatic filtering can all change the result (34, 35). Without harmonized reporting, apparent differences in clinical performance may reflect laboratory workflow rather than biology.

Timing is also important. Resection and ablation can release non-tumor cfDNA through tissue injury and inflammation (20, 21). Sampling too early may dilute ctDNA or create unstable background signals. Sampling too late may miss early postoperative risk information. Published studies have used different windows, including 7–10 days, 30–60 days, and serial follow-up intervals (20, 21, 36, 37).

Clonal hematopoiesis is another major confounder. Low-frequency plasma variants may arise from hematopoietic clones rather than HCC. Matched leukocyte sequencing and careful variant filtering are important, especially for tumor-naive assays (30, 31).

Positivity thresholds are not only technical settings; they are clinical interpretation rules. A threshold for advanced HCC detection may not fit postoperative MRD, and a threshold that triggers repeat testing may not be appropriate for earlier imaging or treatment. In HCC, serial dynamics are likely to be more meaningful than a single cutoff. Persistent positivity, conversion to positivity, or rising molecular burden should carry more weight than an isolated low-level signal.

### 3.8 Practical interpretation of assay choice in HCC-MRD

Assay choice should follow the clinical question. Tumor-informed mutation tracking is compelling when the aim is to detect residual disease from a resected tumor and tissue is available. Tumor-naive panels or methylation assays may be more practical when tissue is unavailable. Methylation and composite cfDNA approaches may add value when low shedding or field-effect biology is a major concern.

No current assay should be used as a standalone treatment trigger in HCC. Positive results should support closer imaging, shorter follow-up, repeat testing, or trial consideration; negative results should be interpreted in relation to baseline detectability, assay sensitivity, tumor features, treatment modality, and liver-field risk.

## 4 Clinical evidence after curative-intent therapy

**Figure 2:**
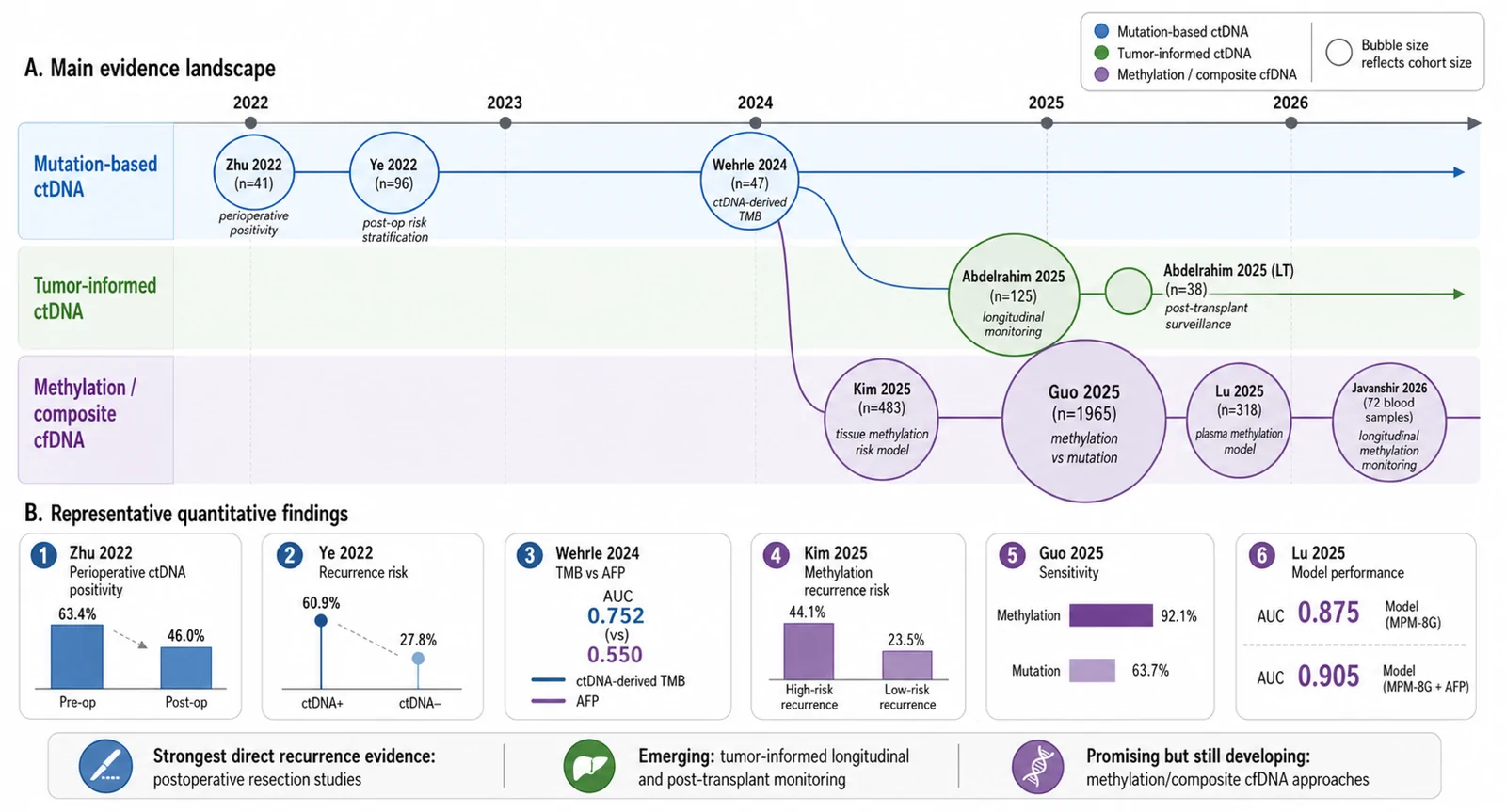
Evidence landscape for ctDNA/cfDNA studies informing recurrence-risk assessment and postoperative surveillance in HCC. **(A)** Representative studies arranged by publication year and assay category, including mutation-based ctDNA, tumor-informed ctDNA, and methylation or composite cfDNA approaches. Bubble size schematically reflects cohort size and is not area-proportional. The panel includes both direct postoperative recurrence-surveillance cohorts and complementary studies that inform recurrence-risk biology or platform sensitivity; these groups should not be interpreted as equivalent levels of evidence. **(B)** Selected descriptive quantitative findings from representative studies. Zhu et al. reported preoperative and postoperative ctDNA positivity rates of 63.4% and 46.0%, respectively (36). Ye et al. reported recurrence rates of 60.9% in ctDNA-positive patients and 27.8% in ctDNA-negative patients after surgery (37). Wehrle et al. reported AUC values of 0.752 for ctDNA-derived tumor mutational burden and 0.550 for AFP in recurrence prediction (39). Kim et al. reported recurrence rates of 44.1% and 23.5% in methylation-defined high-risk and low-risk groups, respectively (32). Guo et al. reported sensitivity values of 92.1% for methylation-based detection and 63.7% for mutation-based detection (23). Lu et al. reported AUC values of 0.875 for the MPM-8G plasma methylation model and 0.905 after combination with AFP (38). These values are shown as study-level examples only and are not directly comparable across platforms, endpoints, sampling windows, or treatment settings. AFP, alpha-fetoprotein; AUC, area under the receiver operating characteristic curve; cfDNA, cell-free DNA; CH, clonal hematopoiesis; CT, computed tomography; ctDNA, circulating tumor DNA; DCP, des-gamma-carboxy prothrombin; HCC, hepatocellular carcinoma; LT, liver transplantation; MDT, multidisciplinary team; MPM-8G, methylation predictive model based on eight genes/miRNAs; MRI, magnetic resonance imaging; MRD, minimal residual disease; MVI, microvascular invasion; post-op, postoperative; pre-op, preoperative; TMB, tumor mutational burden.

Clinical evidence for ctDNA/cfDNA-based surveillance in HCC is growing, but it remains heterogeneous in purpose as well as design. Some studies directly evaluate postoperative recurrence risk after curative-intent therapy, particularly after resection. Others examine longitudinal monitoring in mixed treatment settings, whereas still others inform recurrence-risk biology or platform sensitivity without functioning as dedicated postoperative surveillance cohorts. **Figure 2** summarizes this landscape, and **Supplementary Table 1** provides the study-level context needed to interpret it.

Despite this heterogeneity, several themes recur. Baseline ctDNA detectability often tracks aggressive tumor biology. Postoperative or persistent ctDNA positivity is generally associated with higher recurrence risk in resection cohorts. Serial dynamics appear more informative than a single blood draw. Methylation-based and composite cfDNA approaches may add value when mutation-only tracking is limited by low shedding or by the possibility of *de novo* recurrence, although their direct postoperative surveillance role remains less established. Taken together, the current evidence supports ctDNA as an adjunctive surveillance signal rather than a standalone basis for treatment decisions.

### 4.1 Pre-treatment ctDNA and aggressive tumor biology

Pre-treatment ctDNA provides a molecular baseline before curative-intent therapy. It identifies tumors that shed measurable DNA and makes postoperative results easier to interpret; a postoperative negative result is more informative when the tumor was ctDNA-positive before treatment.

HCC studies link pre-treatment ctDNA with adverse biology. Detectable ctDNA or higher variant allele fraction has been associated with tumor burden, vascular invasion, and recurrence-related outcomes. In Zhu et al., ctDNA was detectable in 63.4% of patients before surgery, and baseline median variant allele frequency independently predicted recurrence-free survival (36).

This information should be viewed as risk enrichment rather than as a substitute for imaging stage, liver function, pathology, or serum biomarkers.

### 4.2 Postoperative ctDNA after resection

The most direct HCC-MRD evidence comes from resection studies, which ask whether persistent or re-emergent tumor-derived DNA after apparent tumor removal identifies patients at high risk of recurrence.

Zhu et al. provided prospective evidence using serial ctDNA profiling after curative liver resection. ctDNA was detected in 63.4% of patients preoperatively and 46.0% postoperatively. Perioperative positivity was associated with shorter recurrence-free survival, and several plasma-detected alterations were linked to shorter time to recurrence (36).

Ye et al. studied postoperative ctDNA in patients after radical resection, with plasma collected 7–10 days after surgery. ctDNA-positive patients had a higher recurrence rate than ctDNA-negative patients. Postoperative ctDNA independently predicted disease-free survival and overall survival, and combining ctDNA with AFP improved postoperative risk stratification (37).

These studies support postoperative ctDNA testing as a risk marker, but their findings should not be overextended. Cohorts were modest, sampling windows varied, and no study has shown that ctDNA-guided intervention improves survival. Postoperative positivity should therefore prompt closer surveillance or trial consideration, not automatic treatment.

### 4.3 Serial monitoring and molecular lead time

Serial monitoring is central to the value of ctDNA. A single postoperative result can be affected by shedding, timing, procedure-related cfDNA release, assay sensitivity, and clonal hematopoiesis. Repeated positivity, conversion to positivity, or rising molecular burden is more likely to reflect clinically meaningful recurrence biology.

Available HCC studies support this principle. Zhu et al. showed that serial ctDNA tracking reflected tumor burden and recurrence risk (36). Abdelrahim et al. also reported the feasibility of longitudinal tumor-informed ctDNA monitoring after curative-intent therapy. Their real-world study included resection and transplantation settings (27).

Molecular lead time remains incompletely standardized because it depends on assay design, baseline shedding, treatment modality, tumor biology, and surveillance schedule. The near-term value of serial ctDNA is risk updating that may justify earlier imaging or shorter follow-up intervals.

### 4.4 Methylation-defined recurrence risk and cfDNA monitoring

Methylation-based studies provide complementary evidence. In HCC, methylation is biologically relevant because epigenetic alterations occur early in hepatocarcinogenesis and may reflect both tumor-derived and field-effect biology.

Kim et al. developed a methylation-based classification system for *de novo* recurrence risk after resection. The study used tissue methylation rather than plasma ctDNA. It remains conceptually important because it focused on *de novo* recurrence and showed that methylation-defined high-risk groups had higher recurrence risk (32).

Javanshir et al. extended this concept to plasma cfDNA methylation. Their study used targeted CpG methylation panels and included longitudinal plasma samples across treatment settings. Plasma methylation signals decreased after resection or transplantation and increased with progression (24).

Large plasma methylation studies also support platform feasibility. Guo et al. found that methylation models outperformed mutation models for HCC detection in a multicenter cohort (23), and Lu et al. reported that a plasma methylation model improved early-stage HCC detection compared with AFP alone (38). These studies are not postoperative MRD trials, but they support the sensitivity of plasma methylation platforms.

Methylation assays may be valuable for low-shedding disease, *de novo* recurrence-risk assessment, and longitudinal monitoring. A positive methylation signal may reflect tumor-derived DNA, field-effect biology, or both, and should be interpreted as recurrence-risk refinement rather than proof of residual disease from the index tumor.

### 4.5 Discordant evidence and the limits of binary positivity

Not all studies support simple binary ctDNA positivity. Wehrle et al. found that identifiable postoperative ctDNA alone was not significantly associated with recurrence-free survival. However, ctDNA-derived tumor mutational burden was associated with shorter recurrence-free survival and higher recurrence risk (39).

This discordance does not invalidate ctDNA surveillance. It shows that signal summarization matters. HCC may shed little DNA, tumor-naive assays may miss relevant variants or detect ambiguous variants, postoperative cfDNA may be affected by timing and tissue injury, and some recurrences may arise from new clones.

The practical message is that persistent positivity, rising burden, and concordance with other risk features should carry more weight than isolated positivity. Negative ctDNA should also be interpreted cautiously, especially in baseline plasma-negative or low-shedding tumors (17).

### 4.6 Evidence synthesis

Overall, current evidence supports the prognostic relevance of ctDNA and cfDNA after curative-intent HCC therapy. Postoperative ctDNA positivity, persistent molecular detectability, higher quantitative burden, and methylation-defined high-risk states are linked to recurrence risk (32, 36, 37, 39). Systematic reviews and meta-analyses support the association between ctDNA positivity and worse survival outcomes, although study heterogeneity remains substantial (40, 41).

The evidence is strongest for risk stratification and weakest for treatment decision-making. No completed prospective HCC trial has shown that ctDNA-guided surveillance or ctDNA-triggered therapy improves survival, so ctDNA should remain an adjunctive surveillance signal outside clinical trials.

## 5 Modality-specific and multimodal interpretation

The meaning of a ctDNA or cfDNA result depends on treatment modality. Resection, ablation, and transplantation differ in tissue availability, procedure-related cfDNA release, liver-field risk, and recurrence pattern.

### 5.1 Resection

Resection provides tumor tissue for pathology and tumor-informed assay design. It also leaves the remaining liver in place. Both residual disease and *de novo* carcinogenesis remain relevant after surgery.

A positive tumor-informed ctDNA result after resection strongly suggests residual clonal disease. This interpretation is stronger when positivity persists or increases over time. A negative result is reassuring only in context. If the tumor was plasma-negative before surgery, postoperative negativity has limited meaning. If a strongly positive baseline signal becomes repeatedly negative, the result supports molecular clearance. It still does not eliminate field-driven recurrence risk.

### 5.2 Ablation

Ablation creates a different problem. Tumor tissue may be unavailable. Local necrosis and inflammation can increase cfDNA. Early imaging may be difficult to interpret because post-ablation changes can mimic or obscure viable tumor.

Serial cfDNA or ctDNA may help in this setting. A persistent or rising signal after an adequate interval could support earlier repeat imaging or multidisciplinary review. Isolated very early positivity should be interpreted cautiously. Methylation-based or composite cfDNA approaches may be useful when tissue is unavailable, but ablation-specific validation is needed (42, 43).

### 5.3 Liver transplantation

Transplantation removes the tumor-bearing liver and the cirrhotic field. This makes post-transplant ctDNA interpretation conceptually cleaner. A patient-specific tumor signal after transplantation is more concerning for residual or recurrent disease than for ongoing native-liver carcinogenesis.

This setting is still complex. Transplant candidates are highly selected. Recurrence events are uncommon but clinically serious. Immunosuppression, explant pathology, AFP dynamics, tumor burden, and selection criteria all affect risk. ctDNA may add dynamic information to established post-transplant models, but it should not replace standard surveillance (28, 29, 44).

### 5.4 Cross-modality surveillance logic

Across treatment modalities, ctDNA is most useful for longitudinal risk updating within a multimodal surveillance system. Baseline status should be established when possible, isolated early results should be interpreted cautiously, unexpected molecular findings should prompt repeat testing, and all results should be interpreted with imaging, AFP/DCP dynamics, pathology, treatment modality, liver function, etiology, and liver disease background.

The emphasis differs by setting: after resection, the key issue is residual disease plus liver-field risk; after ablation, residual viable tumor versus postprocedural change; and after transplantation, recurrent disease in a high-consequence setting.

Conventional biomarkers, imaging, and other liquid biopsy analytes define the clinical context in which ctDNA or cfDNA results should be used.

### 5.5 AFP, AFP-L3, DCP/PIVKA-II, and composite serum models

AFP remains the most widely used serum biomarker in HCC surveillance. Its performance varies by etiology, stage, and postoperative context. Some tumors do not produce AFP. AFP can also be influenced by inflammation or regeneration. AFP-L3 and DCP/PIVKA-II can refine discrimination in selected settings. Composite models such as GALAD may improve blood-based risk assessment (8).

The relevant question is not whether ctDNA should replace AFP or DCP. It is whether ctDNA adds value to surveillance systems that already include imaging and serum markers. ctDNA may provide a molecular relapse signal. AFP and DCP provide broader indicators of tumor activity or burden (9, 33).

### 5.6 Imaging

Imaging remains the anchor of HCC recurrence surveillance because treatment decisions require anatomical localization, intrahepatic extent, extrahepatic spread, vascular invasion assessment, and evaluation of the liver background. ctDNA cannot provide this information.

The realistic role of ctDNA is to adjust imaging strategy. A rising or persistent molecular signal may justify earlier MRI or CT, shorter intervals, or closer multidisciplinary review, whereas a negative molecular result should not replace imaging.

### 5.7 CTCs, exosomes, extracellular RNA, and other liquid biopsy analytes

ctDNA is one part of the liquid biopsy landscape. Circulating tumor cells, exosomes, extracellular RNA, and related analytes provide distinct information about viable dissemination, tumor-host communication, and the liver microenvironment (45–48). Their immediate role in HCC-MRD is less mature than ctDNA/cfDNA, so they are best viewed as complementary tools rather than as the main surveillance framework.

### 5.8 Incremental value of ctDNA in multimodal surveillance

ctDNA is useful only if it improves risk assessment beyond existing tools. Its value is most plausible in high-risk postoperative patients, low-volume residual disease, equivocal imaging, and serial risk updating. A practical model would combine treatment modality, pathology, liver disease background, AFP/DCP dynamics, imaging, and molecular data to identify patients suitable for standard follow-up, intensified surveillance, or trial referral. This is not simply a technical preference. It reflects the fact that genomic analysis alone has not yet solved HCC surveillance because recurrence may arise from residual tumor, intrahepatic spread, or *de novo* carcinogenesis in an injured liver field.

Preliminary clinical studies support this multimodal direction. Ye et al. reported that combining postoperative ctDNA with AFP improved postoperative risk stratification after resection (37). Wehrle et al. found that ctDNA-derived tumor mutational burden showed stronger recurrence discrimination than AFP in an exploratory cohort, whereas binary ctDNA detection alone was less consistent (39). Non-genomic models provide the complementary clinical context: longitudinal biomarker surveillance and time-updated models incorporating tumor burden and AFP dynamics have improved recurrence-risk estimation after resection (9, 33), and AFP, AFP-L3, DCP, and composite serum models such as GALAD may refine blood-based risk assessment (8). After transplantation, clinicopathologic models such as RETREAT also show why tumor burden, AFP, and microvascular invasion remain essential comparators for any molecular marker (44).

This multimodal framing is especially important because ctDNA/cfDNA alone cannot localize recurrence, define resectability, characterize liver reserve, or distinguish all sources of cfDNA release. Molecular positivity should therefore increase attention to conventional surveillance rather than replace it, and molecular negativity should be treated as one reassuring feature among several rather than as permission to relax established follow-up.

## 6 Clinical implementation and ctDNA-guided trial design

**Figure 3:**
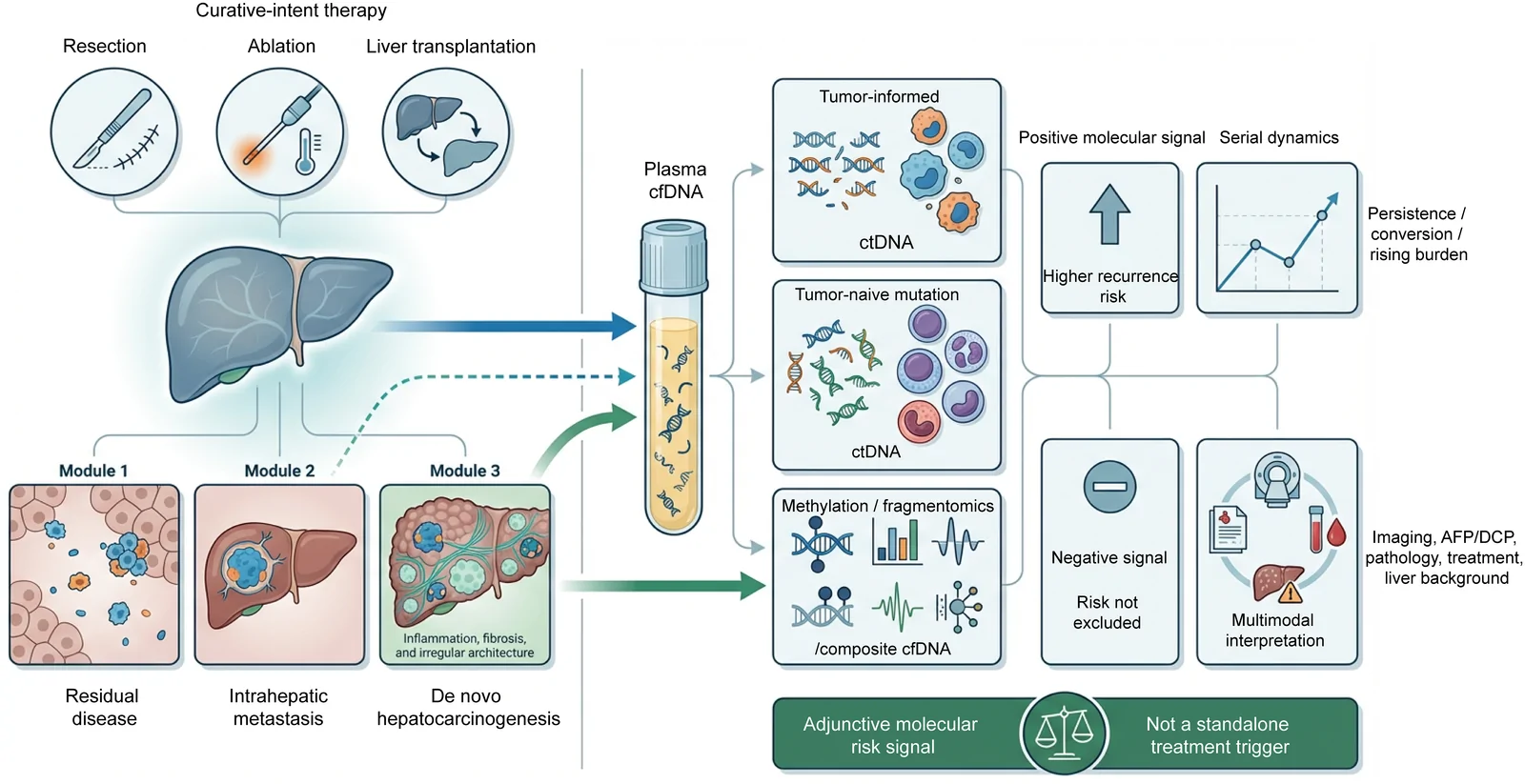
Proposed clinical integration framework for ctDNA/cfDNA-guided surveillance after curative-intent therapy for HCC. This proposed roadmap illustrates how ctDNA/cfDNA could be integrated into recurrence surveillance after curative-intent HCC therapy, including resection, ablation, and liver transplantation. Baseline recurrence risk should be assessed using tumor burden, microvascular invasion, pathology, AFP/DCP, liver function, etiology, and the underlying liver disease background. Assay selection should then be matched to the biological question: tumor-informed ctDNA for patient-specific clone tracking and MRD assessment when tissue is available; tumor-naive mutation-based ctDNA for scalable plasma monitoring when tissue is unavailable, with attention to low tumor fraction and clonal hematopoiesis; and methylation, fragmentomic, or composite cfDNA approaches for broader low-shedding and field-effect risk signals. Longitudinal surveillance should integrate serial blood sampling with MRI or CT, serum biomarkers, and clinical follow-up, with emphasis on signal dynamics rather than isolated binary results. Positive or rising molecular signals may support intensified imaging, repeat testing, and multidisciplinary review, whereas negative or stable signals do not eliminate recurrence risk and should not replace routine surveillance or reduce imaging frequency outside validated protocols. The lower ribbon highlights requirements before routine adoption: prospective validation, standardized sampling, assay-specific thresholds, cost and resource-use assessment, and interventional trials testing whether ctDNA/cfDNA-guided care improves clinical outcomes. AFP, alpha-fetoprotein; AUC, area under the receiver operating characteristic curve; cfDNA, cell-free DNA; CH, clonal hematopoiesis; CT, computed tomography; ctDNA, circulating tumor DNA; DCP, des-gamma-carboxy prothrombin; HCC, hepatocellular carcinoma; LT, liver transplantation; MDT, multidisciplinary team; MPM-8G, methylation predictive model based on eight genes/miRNAs; MRI, magnetic resonance imaging; MRD, minimal residual disease; MVI, microvascular invasion; post-op, postoperative; pre-op, preoperative; TMB, tumor mutational burden.

Any clinical use of ctDNA in HCC must be framed as adaptive surveillance rather than binary treatment triggering. The practical question is not only when to sample, but how a molecular result should modify imaging timing, repeat testing, and multidisciplinary review. **Figure 3** therefore presents a proposed implementation framework, not a validated management algorithm.

### 6.1 Baseline molecular profiling

Baseline profiling before curative-intent therapy establishes whether a tumor sheds a measurable molecular signal and can guide subsequent interpretation (25).

When tissue is available, tumor sequencing can support tumor-informed assay design. When tissue is unavailable, plasma methylation or tumor-naive cfDNA approaches may be practical, but their interpretation should remain cautious.

### 6.2 Early post-treatment reassessment

Early postoperative or postprocedural testing can provide an initial molecular reassessment, but the optimal timing remains unsettled. Surgery-related cfDNA release and sample-processing effects complicate very early interpretation, and published studies have used windows from 7–30 days to 30–60 days after surgery (20, 21, 37). Single early positive or negative results should therefore be interpreted in relation to baseline detectability, pathology, serum biomarkers, and imaging context.

### 6.3 Serial risk updating during follow-up

Serial monitoring is more informative than one isolated sample. Persistently negative results may support continuation of standard surveillance, but they should not justify less frequent imaging outside a prospective de-escalation protocol. By contrast, persistent positivity, conversion to positivity, or rising molecular burden may justify reassessment of imaging timing and follow-up intensity (36). Reports should state assay type, tumor-informed or tumor-naive design, quantitative burden when available, limit of detection, leukocyte filtering status, sampling date relative to treatment, and prior longitudinal results.

### 6.4 How ctDNA positivity should change management

ctDNA positivity should not directly trigger treatment outside clinical trials. Its realistic role is to support intensified imaging, shorter intervals, repeat molecular confirmation, multidisciplinary review, and trial consideration (15, 41). These actions remain surveillance adaptations rather than evidence-based treatment mandates. **Table 2** summarizes possible scenarios, but clinical implementation requires prospective trials that prespecify how molecular results will change surveillance or treatment pathways, including rules for discordant molecular and imaging findings.

**Table 2:** Proposed clinical scenarios for interpreting ctDNA/cfDNA findings during post-treatment surveillance in HCC.

Clinical scenario	ctDNA/cfDNA finding	Possible interpretation	Suggested clinical response	Evidence level
Baseline ctDNA positive before resection	Detectable tumor-derived signal before treatment	Higher-risk biology; measurable plasma signal available for follow-up	Document baseline status; plan early post-treatment reassessment; integrate with pathology and imaging	Moderate
Early postoperative ctDNA negative	No detectable ctDNA soon after treatment	Molecular clearance or low-shedding disease; not equivalent to no recurrence risk	Continue standard imaging plus AFP/DCP; interpret in light of baseline signal and assay sensitivity; do not reduce imaging frequency outside a validated de-escalation protocol	Low to moderate
Persistent postoperative ctDNA positive	Repeated detectable ctDNA after treatment	Residual disease or high-risk biology	Consider intensified imaging, shorter follow-up interval, multidisciplinary review, and trial enrollment	Moderate
Conversion from negative to positive during follow-up	Newly detectable ctDNA after prior negativity	Emerging recurrence signal or rising residual disease burden	Repeat ctDNA confirmation; consider earlier cross-sectional imaging and interval shortening if confirmed or clinically concordant	Low to moderate
Rising molecular burden with negative imaging	Increasing quantitative ctDNA/cfDNA abnormality without radiologic recurrence	Possible molecular lead time before visible recurrence	Consider repeat assay, earlier MRI/CT, and multidisciplinary review; weigh false-positive risk and resource use	Low
High-risk methylation signature	Methylation-defined high-risk state	Recurrence-prone biology, including *de novo* carcinogenesis risk	Consider closer surveillance and integration with clinicopathologic risk models; do not treat as standalone proof of residual disease	Moderate
Post-ablation molecular positivity	Detectable ctDNA/cfDNA signal after ablation	Residual viable disease or early recurrence risk	Consider earlier imaging and shorter surveillance interval	Low
Post-transplant molecular positivity	Detectable ctDNA after transplantation	Possible recurrent disease in a high-consequence setting	Consider prompt confirmatory imaging, repeat assay, and multidisciplinary review; trial consideration where available	Low

AFP, alpha-fetoprotein; DCP, des-gamma-carboxy prothrombin; CT, computed tomography; MRI, magnetic resonance imaging. This table presents a proposed interpretation framework and should not be read as practice-guideline-level treatment advice. Evidence levels are narrative summaries rather than formal guideline grades. Because most positive molecular scenarios imply additional testing or earlier imaging, cost-effectiveness and downstream resource use should be evaluated prospectively. Negative results should not currently support reduced imaging frequency outside validated protocols.

### 6.5 Enrichment of high-risk patients for adjuvant trials

One promising use of ctDNA is trial enrichment. Patients with persistent postoperative positivity, high molecular burden, or high-risk methylation profiles may have higher recurrence risk than unselected patients (15, 16). Molecular enrichment could improve trial efficiency in adjuvant HCC studies, but thresholds must be validated and predefined.

### 6.6 ctDNA clearance and conversion as dynamic endpoints

ctDNA dynamics may also serve as trial endpoints. Molecular clearance, persistent positivity, and conversion from negative to positive could provide pharmacodynamic or surveillance information (15, 49).

These endpoints require caution. Molecular clearance may reflect reduced burden, timing, assay sensitivity, or low shedding; molecular conversion may precede imaging but does not localize disease. Molecular endpoints should therefore complement recurrence-free survival, overall survival, imaging response, and safety endpoints.

### 6.7 Trial designs needed before clinical adoption

Before routine adoption, HCC needs prospective trials with predefined sampling schedules, interpretation rules, and action algorithms. These trials should compare ctDNA-guided surveillance with realistic standard care and define the clinical response to positive, negative, and discordant molecular signals (11, 50).

Possible designs include ctDNA-informed surveillance intensity, adjuvant trials enriched for ctDNA-positive patients, and early imaging triggered by molecular conversion. The action plan must be specified before the trial starts and should include safety, quality-of-life, and resource-use endpoints.

### 6.8 Resource use, cost, and de-escalation

From a health-system perspective, the main cost consequence of cfDNA-guided surveillance is not the blood draw alone but the downstream pathway it creates. A positive, rising, or discordant result usually leads to repeat testing, earlier MRI or CT, shorter intervals, and multidisciplinary review. This may increase short-term costs compared with guideline-based imaging and serum-marker follow-up, especially if false-positive or biologically ambiguous signals are common. Conversely, economic benefit would require that earlier molecular detection leads to salvageable recurrence, trial enrollment, avoidance of ineffective treatment, or other measurable outcome gains. Direct HCC-specific cost-effectiveness data for post-treatment cfDNA-guided recurrence surveillance are not yet available; extrapolation from colorectal cancer shows that economic value is highly scenario-dependent and must incorporate assay cost, positivity rate, imaging utilization, adjuvant-treatment decisions, survival benefit, and quality-of-life effects (52, 53). Economic modeling of conventional HCC surveillance also shows that value depends on interval, adherence, and early detection assumptions, reinforcing the need to test any molecular add-on against realistic standard-care pathways (54).

At present, negative cfDNA/ctDNA findings should not be used to reduce the frequency of standard HCC follow-up or imaging outside a prospective protocol. A negative result may be more reassuring when the index tumor was ctDNA-positive at baseline, the assay is analytically informative, and serial samples remain negative. Even then, it should support standard surveillance rather than de-escalation because recurrence may be low shedding, below the limit of detection, or *de novo* from the liver field. Future trials should therefore prespecify escalation rules for positive results, de-escalation rules for persistently negative results, economic endpoints, and safety monitoring.

## 7 Limitations, reporting standards, and future directions

Several limitations remain. Recurrence biology is heterogeneous; low tumor fraction and variable shedding reduce sensitivity in the setting where molecular surveillance is most needed; and sample handling, postoperative cfDNA kinetics, and clonal hematopoiesis can confound low-frequency signals (51).

Study heterogeneity is another major problem. Published reports vary in population, liver disease background, treatment modality, assay class, laboratory workflow, sampling window, threshold, and recurrence definition (40, 41). The field is increasingly able to identify high-risk patients, but it is less mature in defining what clinicians should do after positive, negative, or discordant results.

### 7.1 Biological limitations

Biologically, tumor-informed assays may detect residual clonal disease but miss multicentric *de novo* tumors, whereas methylation assays may capture high-risk field biology without proving persistence of malignant cells from the index tumor. Interpretation must therefore remain context-dependent.

### 7.2 Technical and analytical limitations

Technical limitations span the pre-analytical, analytical, and post-analytical pathway. Low tumor fraction, limited cfDNA input, sequencing errors, sample-processing variation, and clonal hematopoiesis can all affect ctDNA interpretation. Cross-study comparison is further limited by inconsistent reporting of blood tubes, processing intervals, centrifugation protocols, storage conditions, plasma volume, cfDNA input, molecular barcoding, duplicate suppression, variant-calling thresholds, and leukocyte filtering. Analytical validation should report limit of detection, reproducibility, specificity, linearity, error suppression, and assay failure or non-informative rates in low-input samples (19, 34, 51). Key reporting elements for HCC ctDNA-MRD studies are summarized in **Box 1**.

Box 1 **Proposed reporting checklist for HCC ctDNA-MRD studies**

- Patient population and HCC etiology.
- Liver disease background and degree of cirrhosis.
- BCLC stage or equivalent staging system.
- Treatment modality: resection, ablation, transplantation, or combined treatment.
- Pathological risk factors, including microvascular invasion and margin status.
- Assay platform, blood collection tube, processing interval, plasma separation, and storage conditions.
- Panel size, target content, and covered genes, loci, or genomic regions.
- Tumor-informed or tumor-naive design.
- Plasma volume and cfDNA input amount.
- Limit of detection.
- Matched leukocyte sequencing or other clonal hematopoiesis filtering strategy.
- Sampling time points relative to treatment.
- Predefined positivity threshold, molecular burden metric, and quantitative reporting method.
- Assay failure rate and criteria for an analytically non-informative negative result.
- Imaging comparator.
- AFP, AFP-L3, or DCP comparator.
- Definition of recurrence.
- Molecular lead time.
- Predefined management action after positive, negative, and discordant assays.
- Follow-up duration.
- External validation cohort.
- Economic and resource-use endpoints if surveillance intensity is changed.

Post-analytical standardization is equally important. Reports should state panel size and content, covered driver genes or CpG loci, tumor fraction or molecular-burden metrics, raw and filtered variant counts when relevant, clonal hematopoiesis filtering, prespecified positivity and conversion thresholds, and whether a negative result is analytically informative. This is particularly important for tumor-naive panels and methylation assays because platforms differ in target selection, bioinformatic pipelines, and thresholds. Without this information, it is difficult to compare assay performance or translate a positive or negative result into a surveillance action.

### 7.3 Clinical-action limitations

Clinically, even accurate risk identification does not define the best response. More frequent imaging may detect recurrence earlier, but survival benefit and cost-effectiveness have not been proven. Adjuvant therapy for ctDNA-positive patients is attractive, but toxicity, liver function, competing risks, and uncertain thresholds must be considered. For now, ctDNA is best used for trial enrichment and surveillance adaptation.

### 7.4 Future perspectives

The most realistic future direction is risk-adapted surveillance rather than universal ctDNA monitoring for every treated HCC patient. ctDNA or broader cfDNA features should be used where they add value over existing care, with methylation and composite cfDNA assays particularly relevant to low-shedding disease and field-effect biology (24).

Progress will depend on shared language for postoperative sampling, laboratory workflow, threshold reporting, serial positivity, molecular lead time, and management response. Future studies should compare ctDNA-guided surveillance with realistic standard care, including resource use and patient-centered outcomes.

The field should also move toward integrated models that combine molecular dynamics with imaging, AFP/DCP trends, pathology, treatment modality, liver function, and etiology.

The endpoint should be patient benefit. Earlier molecular detection matters only if it leads to better outcomes, safer surveillance, better trial enrollment, or more timely treatment of actionable recurrence. Future studies should ask not only whether ctDNA predicts recurrence, but whether acting on ctDNA improves care.

## 8 Conclusion

ctDNA and broader cfDNA approaches provide a compelling framework for MRD assessment and recurrence-risk surveillance after curative-intent therapy for HCC. Their clearest current role is adjunctive risk stratification and serial risk updating, particularly after resection, where the direct postoperative evidence base is most developed. The evidence for ablation and liver transplantation is promising but less mature.

The central challenge is HCC-specific recurrence biology. Relapse may reflect residual malignant disease, occult intrahepatic spread, or multicentric *de novo* hepatocarcinogenesis in a chronically injured liver. Tumor-informed ctDNA is best suited to tracking residual clonal disease, whereas methylation-based and composite cfDNA approaches may capture low-shedding disease and broader recurrence-prone liver-field biology. These signals are complementary, but they should not be interpreted as interchangeable.

At present, ctDNA should be integrated with imaging, AFP/DCP, pathology, treatment modality, and liver disease background. It should not be used as a standalone decision trigger. Positive or rising molecular signals may justify closer surveillance, earlier imaging, repeat testing, multidisciplinary review, or trial consideration, whereas negative results do not eliminate recurrence risk and should not currently reduce standard imaging frequency. The next step for the field is not simply to refine prediction, but to determine through HCC-specific prospective studies whether ctDNA-guided care improves clinically meaningful outcomes in a standardized and cost-conscious manner.
