# Supplementary material for: Circulating tumor DNA for minimal residual disease and recurrence surveillance in hepatocellular carcinoma: current evidence and a translational roadmap
Source: Front Oncol. 2026 Sep 1;16:1920559. doi: 10.3389/fonc.2026.1920559 (PMC13575990; doi:10.3389/fonc.2026.1920559)
Supplement: Supplementary file 1 [file Table1.docx]

# **Supplementary Table 1. Representative clinical and assay-development studies informing ctDNA/cfDNA-based recurrence-risk assessment and surveillance in HCC**

Note: DFS, disease-free survival; OS, overall survival; RFS, recurrence-free survival; VAF, variant allele frequency; HBV, hepatitis B virus; qMSP, quantitative methylation-specific PCR; TCGA, The Cancer Genome Atlas. Included studies vary in treatment setting, assay purpose, and endpoint; not all are direct postoperative recurrence-surveillance cohorts.

| **Study** | **Year** | **Design** | **Patients** | **Treatment setting** | **Assay type** | **Sampling time points** | **Endpoint** | **Key findings** | **Main limitations** |
| --- | --- | --- | --- | --- | --- | --- | --- | --- | --- |
| Zhu et al. | 2022 | Prospective single-center study | 41 | Curative liver resection | Serial ctDNA targeted NGS with matched tissue sequencing | Preoperative and postoperative serial blood sampling | Early recurrence, time to recurrence, recurrence-free survival | Preoperative ctDNA was detected in 63.4% and postoperative ctDNA in 46.0%. Persistent perioperative positivity and higher baseline VAF were associated with worse recurrence outcomes. | Small cohort; single center; limited recurrence events; resection-only population. |
| Ye et al. | 2022 | Observational postoperative prognostic study | 96 | Radical resection for primary HCC | Hybrid-capture NGS using tissue and postoperative plasma panels with matched buffy coat filtering | Postoperative plasma collected 7–10 days after surgery | Disease-free survival, overall survival, recurrence risk stratification | Postoperative ctDNA positivity was associated with higher recurrence and independently predicted DFS and OS. Combining ctDNA with AFP improved risk stratification. | Single postoperative time point; HBV-dominant cohort; no ctDNA-guided intervention. |
| Wehrle et al. | 2024 | Retrospective single-institution study | 47 with postoperative ctDNA testing | Curative-intent surgery, mainly resection | Commercial tumor-naive ctDNA assay and ctDNA-derived tumor mutational burden | Preoperative, postoperative, and serial institutional testing; main analysis used postoperative samples | Recurrence, recurrence-free survival, AFP comparison | Identifiable postoperative ctDNA alone was not significantly associated with RFS, whereas ctDNA-derived TMB was associated with shorter RFS and higher recurrence risk. | Small retrospective cohort; exploratory cutoff; heterogeneous treatment context; no interventional validation. |
| Abdelrahim et al. | 2025 | Retrospective real-world tumor-informed ctDNA study | 125 | Curative-intent therapy including resection and liver transplantation | Personalized tumor-informed ctDNA assay | Longitudinal serial plasma monitoring | Recurrence/progression risk and monitoring | Serial tumor-informed ctDNA monitoring supported early recurrence detection and longitudinal risk clarification after curative-intent therapy. | Retrospective design; heterogeneous treatments; no predefined ctDNA-guided management algorithm. |
| Abdelrahim et al. | 2025 | Retrospective post-transplant tumor-informed ctDNA surveillance study | 38 | Liver transplantation for primary liver cancers including HCC | Personalized tumor-informed ctDNA assay | Serial post-transplant surveillance with standard follow-up | Post-transplant recurrence surveillance | Demonstrated feasibility of tumor-informed ctDNA monitoring after transplantation and supported ctDNA as an adjunctive high-risk surveillance signal. | Small cohort; mixed HCC and cholangiocarcinoma population; early feasibility evidence. |
| Kim et al. | 2025 | Retrospective tissue methylation cohort with validation | 483 total across discovery, validation, and TCGA cohorts | Post-resection HCC with focus on de novo recurrence | Tissue DNA methylation profiling and marker validation | Surgical tissue-based risk assessment with postoperative imaging follow-up | De novo recurrence risk and recurrence-free survival | Methylation-defined high-risk groups had higher de novo recurrence risk, supporting epigenetic recurrence-risk classification. | Tissue-based rather than plasma-based; retrospective; not direct MRD detection. |
| Javanshir et al. | 2026 | Translational methylation assay study with plasma cohort | Tissue validation datasets plus plasma cohort | Mixed settings including resection, transplantation, Y-90, and systemic therapy | Targeted cfDNA methylation panels | Longitudinal plasma sampling before and after treatment | Diagnosis, prognosis, tumor burden monitoring, progression assessment | Plasma methylation signals decreased after resection or transplantation and increased with progression, supporting dynamic monitoring potential. | Small plasma cohort; mixed treatment settings; not a dedicated postoperative MRD trial. |
| Guo et al. | 2025 | Multicenter clinical assay development study | 1,965 participants | HCC detection and high-risk monitoring | Parallel plasma cfDNA mutation and methylation profiling | Cross-sectional plasma sampling | HCC detection and early-stage detection | Methylation models outperformed mutation models for HCC detection and supported feasibility of plasma methylation platforms. | Detection-focused, not recurrence-specific; no postoperative serial MRD assessment. |
| Lu et al. | 2025 | Plasma methylation assay study | 318 plasma samples | HBV-related HCC diagnosis and surveillance-relevant detection | qMSP-based plasma methylation model with AFP comparison | Pretreatment plasma samples | Diagnostic discrimination and early-stage detection | Methylation model improved detection compared with AFP alone and performed well in early-stage HCC. | Detection-focused; HBV-related cohort; not recurrence-specific. |
